# An Address Delivered before the Monroe County Medical Society, at Its Annual Meeting, in Rochester, N. Y.

**Published:** 1846-06-01

**Authors:** 


*An Address delivered before the Monroe County Medical Society, at its Annual Meeting, in Rochester, N. Y., by* E. W. Armstrong, M. D. *President of the Society.*

The subject of this address is "*The Claims of the Medical Profession.*"

Dr. Armstrong enters into a candid consideration of the circumstances which entitle the medical profession to public confidence and support. He freely expresses his dissatisfaction with its present condition as respects the medical, and other attainments of a large proportion of its members. In this, he goes nearly as far as Dr. Davis, whose positions have called forth the ire of Prof. Paine. Dr. Armstrong had better take care or he will get a shot from the defender of the medical profession of the United States!

The means of elevating the profession, in public estimation, are treated of. He thinks it vain to look for legislative encouragement and aid, but that we must depend upon ourselves. The address is well written, and is replete with sound and dignified sentiments. In reading it, we marked several passages for quotation, but the length to which our analysis of Prof. Hun's paper extended, precludes their publication in the present number.

